# Relationship between Vagal Tone, Cortisol, TNF-Alpha, Epinephrine and Negative Affects in Crohn’s Disease and Irritable Bowel Syndrome

**DOI:** 10.1371/journal.pone.0105328

**Published:** 2014-09-10

**Authors:** Sonia Pellissier, Cécile Dantzer, Laurie Mondillon, Candice Trocme, Anne-Sophie Gauchez, Véronique Ducros, Nicolas Mathieu, Bertrand Toussaint, Alicia Fournier, Frédéric Canini, Bruno Bonaz

**Affiliations:** 1 Grenoble Institut des Neurosciences (GIN), Centre de Recherche INSERM 836 Equipe : Stress et Interactions Neuro-Digestives (EA3744), Université Joseph Fourier, Grenoble, France; 2 Département de Psychologie, Université de Savoie, Chambéry, France; 3 Laboratoire Interuniversitaire de Psychologie: Personnalité, Cognition, Changement social (LIP/PC2S), Université de Savoie, Chambéry, France; 4 Laboratoire de Psychologie Sociale et Cognitive (LAPSCO, CNRS UMR6024), Université Blaise Pascal, Clermont-Ferrand, France; 5 Institut de Biologie, Centre Hospitalo-Universitaire de Grenoble, Grenoble, France; 6 Clinique Universitaire d’Hépato-Gastroentérologie, Centre Hospitalo-Universitaire de Grenoble, Grenoble, France; 7 Laboratoire TIMC/TheREx UMR 5525, Université Joseph Fourier, Grenoble, France; 8 Unité de Neurophysiologie du Stress, Institut de Recherche Biomédicale des Armées (IRBA), Brétigny-sur-Orge, France; 9 Ecole du Val de Grâce, Paris, France; University of Chicago, United States of America

## Abstract

Crohn’s disease (CD) and irritable bowel syndrome (IBS) involve brain-gut dysfunctions where vagus nerve is an important component. The aim of this work was to study the association between vagal tone and markers of stress and inflammation in patients with CD or IBS compared to healthy subjects (controls). The study was performed in 73 subjects (26 controls, 21 CD in remission and 26 IBS patients). The day prior to the experiment, salivary cortisol was measured at 8∶00 AM and 10∶00 PM. The day of the experiment, subjects completed questionnaires for anxiety (STAI) and depressive symptoms (CES-D). After 30 min of rest, ECG was recorded for heart rate variability (HRV) analysis. Plasma cortisol, epinephrine, norepinephrine, TNF-alpha and IL-6 were measured in blood samples taken at the end of ECG recording. Compared with controls, CD and IBS patients had higher scores of state-anxiety and depressive symptomatology. A subgroup classification based on HRV-normalized high frequency band (HFnu) as a marker of vagal tone, showed that control subjects with high vagal tone had significantly lower evening salivary cortisol levels than subjects with low vagal tone. Such an effect was not observed in CD and IBS patients. Moreover, an inverse association (r = −0.48; p<0.05) was observed between the vagal tone and TNF-alpha level in CD patients exclusively. In contrast, in IBS patients, vagal tone was inversely correlated with plasma epinephrine (r = −0.39; p<0.05). No relationship was observed between vagal tone and IL-6, norepinephrine or negative affects (anxiety and depressive symptomatology) in any group. In conclusion, these data argue for an imbalance between the hypothalamus-pituitary-adrenal axis and the vagal tone in CD and IBS patients. Furthermore, they highlight the specific homeostatic link between vagal tone and TNF-alpha in CD and epinephrine in IBS and argue for the relevance of vagus nerve reinforcement interventions in those diseases.

## Introduction

Crohn’s disease (CD) is an inflammatory bowel disease (IBD) characterized by a chronic abnormal mucosal immune response with periods of remission of unpredictable duration alternating with acute episodes of flare [1], [2]. Irritable bowel syndrome (IBS) is a highly prevalent functional gastrointestinal disorder characterized by abdominal pain and discomfort associated with altered bowel habits [3]. Both pathologies involve brain-gut interaction perturbations and are strongly influenced by narrow interactions between biological and psychosocial factors, and thus considered as bio-psychosocial diseases [4]–[8]. High perceived stress, negative affects such as anxiety, depression and an imbalanced autonomic nervous system (ANS) are common features in CD and IBS [7], [9], [10]. The neuroendocrine communication between the brain and the gut is mediated by the parasympathetic and sympathetic branches of the ANS, and by the hypothalamus-pituitary-adrenal (HPA) axis (Bonaz and Bernstein, 2013 for review). These regulatory systems, as a part of the allostatic network, are interrelated and functionally coupled to adapt physiological responses to external and/or internal challenges ensuring homeostasis and promoting health [11]–[13]. Specifically, the parasympathetic nervous system plays a major role in gastrointestinal homeostasis [14] and is involved in physiological and psychological flexibility in reaction to stress [15], [16], emotional regulation, and stress recovery [17], [18]. Furthermore, the parasympathetic nervous system, through the vagus nerve, modulates the production of pro-inflammatory cytokines such as TNF-alpha [19] through both vagal afferents and efferents activating respectively the HPA axis and the cholinergic anti-inflammatory pathway [9], [20], [21]. TNF-alpha is a key pro-inflammatory cytokine involved in CD and anti-TNF therapy is currently the gold standard in the treatment of IBD patients [22]. The vagus nerve is also combined with the HPA axis and under physiological conditions a balance is observed between the parasympathetic nervous system and the HPA axis [23]. This reflects an adapted homeostatic regulation by coupling high vagal tone to low cortisol level. However, in chronic diseases such as alcoholism, where the parasympathetic tone is dramatically blunted, this coupling is altered [24] reflecting an impaired inhibitory control of the HPA axis and an allostatic load as defined by McEwen [25]. An autonomic imbalance with a sympathetic dominance has been described in IBD and IBS [10], [26] and should logically have an impact on the HPA axis regulation and thus on catecholamines and pro-inflammatory cytokines levels such as TNF-alpha or IL-6. However, little is known about the nature of the relationship between the vagal tone and the HPA axis in these pathologies and even less with catecholamines and pro-inflammatory cytokines. This raises the question of the correlation, in CD or IBS patients, between the resting vagal tone, which could be considered as a functional parasympathetic fingerprint, on the one hand, and cortisol, catecholamines and pro-inflammatory cytokines levels on the other hand.

Consequently, the principal aim of this study was to examine this functional coupling. If the ANS and the HPA axis are functionally uncoupled in CD and IBS, then we should find no relation between vagal tone and cortisol levels in patients while a high vagal tone will be associated to a low cortisol level (and conversely) in controls. Furthermore, we hypothesized that negative affects (anxiety and depressive symptomatology), catecholamines and cytokines levels were dependent on vagal tone in CD and IBS patients but not in controls. For this purpose, heart rate variability (HRV), an index of the parasympathetic nervous system activity, was measured at rest in control healthy subjects, CD patients in remission and IBS patients. Then, a cluster analysis was performed in order to compare, between the low and high vagal tone subgroups, the levels of cortisol, TNF-alpha, IL-6, epinephrine, norepinephrine and negative affects.

## Materials and Methods

### Subjects and Ethics Statement

The study was performed in agreement with the Declaration of Helsinki and the guidelines of Good Clinical Practice and was approved by the Ethic Committee of the Grenoble Faculty of Medicine and Hospital (ref: 08-CHUG-23, ClinicalTrials.gov Identifier: NCT01095042). Written informed consent was obtained from each participant. White subjects, aged 18–60 years, were prospectively recruited between September 2009 and October 2011. CD and IBS patients were recruited in our Department of Gastroenterology while age and sex-matched healthy subjects were recruited by the Grenoble INSERM Clinical Investigation Centre (CIC).

### Criteria for Inclusion

#### Crohn’s Disease (CD) patients

CD patients were selected according to their phenotype as defined by the Montreal classification [27]. CD patients with isolated ano-perineal or upper digestive tract lesions were not eligible. CD activity was evaluated by the Harvey–Bradshaw index (HBI) [28] and patients with an HBI<4 on inclusion were considered in clinical remission. The endoscopic, contrast-enhanced ultrasound and biologic explorations (CRP<5 mg/l) showed that all patients were under mucosal healing and/or parietal healing under their current treatment. Patients were included only if they had a stable dose of *i) 5-aminosalicylates* for at least 2 weeks, *ii) immunosuppressives* for at least 12 weeks, and *iii) biological therapy* (e.g., anti-TNF-alpha) for at least 8 weeks.

#### Irritable Bowel Syndrome (IBS) patients

Patients were selected according to Rome II criteria [29]: at least 12 weeks, not necessarily consecutive, in the preceding 12 months of abdominal discomfort or pain with two out of the three following features: 1) relieved with defecation; and/or 2) onset associated with a change in frequency of stool; and/or 3) onset associated with a change in form (appearance) of stool. The lack of organicity for patient’s symptoms was assumed through: *i)* a negative physical examination; *ii)* a normal colonoscopy performed within the last five years with normal biopsies (i.e., absence of microscopic colitis); *iii)* normal limited laboratory evaluations with a lack of inflammation (i.e., erythrocyte sedimentation rate, C-reactive protein), anaemia, infection (complete blood cell count) and endocrine or metabolic disturbances (i.e., thyroid stimulating hormone, chemical analysis) as well as the absence of IgA anti-transglutaminase (without IgA deficiency).

### Criteria for Exclusion

Patients were excluded from the study if: *(i)* they had past or present medical conditions complicated by autonomic dysfunction (e.g., peripheral neuropathy, diabetes, vagotomy, dysthyroidism, amyloidosis, asthma, heart failure, renal insufficiency, alcoholism), *(ii)* they were under medication susceptible to modify the ANS (e.g., anticholinergics, antiarrhytmics, alpha or beta blocking agents, antibiotics). Patients with previous abdominal surgery, except appendectomy and/or cholecystectomy, were excluded from the study.

### Experimental Design

**Figure 1:**
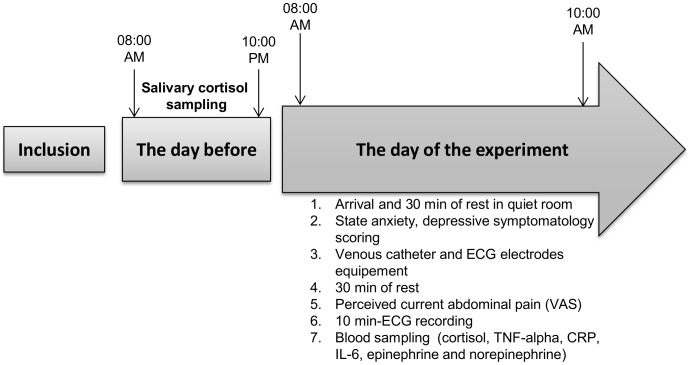
The experimental design.

All patients underwent an interview concerning their history (disease duration, extent, extra-intestinal manifestations, course, current and past therapies, medications) and a physical examination to determine their inclusion in the study according to the inclusion-exclusion criteria. After information and consent, subjects were enrolled and an appointment was fixed. As shown in figure 1, the day before the experiment, salivary cortisol was measured at 08∶00 AM and 10∶00 PM at home. Participants were asked to have a light breakfast on the morning of their running session. On their arrival in our department (8∶00 AM), each participant was oriented to a quiet room to sit and relax during 30 min in a comfortable chair. After explanations on the running of the session, participants completed questionnaires for state-anxiety (State-Trait Anxiety Inventory; STAI) [30] and depressive symptomatology (Center for Epidemiologic Studies-Depression Scale; CES-D) [31], [32]. They were then equipped with electrodes for electrocardiogram (ECG) recording and a venous catheter for blood sampling. After a resting period of 30 min, participants were asked to evaluate their current visceral perception through a visual analogic scale (VAS) measuring the intensity of perceived abdominal pain (0: no perceived pain; 10: the maximum perceived pain), then the electrocardiogram (ECG) was recorded for 10 minutes. During this recording period, a technician carefully observed the optimal conditions to ensure that the recording was free of body movements, conversations and any subjective discomfort. Experimental sessions were always performed between 08.00 AM and 10.00 AM to avoid any influence of circadian variations. Catecholamines (epinephrine, norepinephrine), pro-inflammatory cytokines (TNF-alpha, IL-6), cortisol and C-reactive protein (CRP) were measured in blood samples collected at the end of the resting ECG.

### Parasympathetic Assessment: Power spectral analysis of Heart Rate Variability (HRV)

ANS activity was explored using HRV as a reliable and non-invasive method [33]. Initially described by Akselrod [34] to explore cardiovascular control, this tool is now commonly used in gastrointestinal physiology to assess autonomic imbalance related to digestive autonomic regulation [10], [35]. ECG signal was acquired through electrodes placed on each wrist. HRV analysis was performed using specific software (Heart Rhythm Scanner, Biocom Technologies, USA). First, QRS complexes were automatically classified. Ectopic or abnormal QRS complexes were visually detected in the software and removed. The signal was then carefully checked and remaining abnormalities were manually removed. Then, a standard spectral analysis was applied on inter-beat intervals using a Fast Fourier Transformation (FFT) according to the standards of measurement of the Task Force on HRV [36]. The following parameters were calculated: *(i)* Total Power (TP) corresponds to the spectral power density in the range of 0 to 0.40 Hz. It was considered as the net effect of all physiological mechanisms contributing to HRV; *(ii)* High Frequency power spectrum (HF, from 0.15 to 0.40 Hz, msec^2^) reflected for 90% parasympathetic tone fluctuations caused by respiratory sinus arrhythmia; [37], [38] *(iii)* Low Frequency power spectrum (LF, from 0.04 to 0.15 Hz, msec^2^) at rest in sitting position, is explained by parasympathetic tone for at least 50% and sympathetic tone for 25%. This HRV component is related to baroreflex modulation [38] *(iv)* Very Low Frequency power spectrum (VLF, from 0.0033 to 0.04 Hz, msec^2^) represented various negative emotions or worries in short time recording [39] and various long term endocrine regulations such as renin-angiotensin system and thermoregulation [36], [40]. LF and HF variables were also expressed in normalized units: normalized HF [HFnu = HF/(TP–VLF)] and normalized LF [LFnu = LF/(TP–VLF)], respectively. This calculation minimized the effect of changes in Very Low Frequency power on LF and HF power and emphasized the changes in sympathetic or parasympathetic regulation. *(v)* Lastly, LF/HF ratio was calculated as a global marker of the autonomic balance.

### Cytokines Measurement

Interleukin-6 and TNF-alpha were evaluated by the Randox Biochip Array technology (Randox Laboratories, Roissy-en-France). This miniaturized ELISA-based technic allows simultaneous quantitative detection of multiple cytokines from a patient low volume single sample. The array used in this study is the Cytokine Array I, which is coated with antibodies against 12 cytokines. Briefly 100 µl of EDTA plasma or standards were added in each well of the biochip and were incubated for 1 hour at 37°C at 370 rpm. Biochip was quickly washed twice with 350 µl of wash buffer, and 4 more washings with a 2-minute soaking step were performed. Then 300 µl of HRP-conjugate antibodies were added and incubated for 1 hour at 37°C at 370 rpm. Washings were realized as previously described and the biochip was briefly air dried. The two components of the signal reagent, luminol and peroxide, were mixed in a ratio of 1∶1 and 250 µl were added per well. Signal reading was performed on the Randox Evidence Investigator device, after incubation of the biochip for 2 minutes in the dark. Captured RLU were converted into concentration of cytokines using the 9-point calibration curves run in parallel for each cytokine.

### Salivary Cortisol Measurements

Saliva was collected on Salivette (Sarstedt, Marnay, France) the day before the experiment at 8∶00 AM and 10∶00 PM and stored at −20°C until analysis. Cortisol was evaluated by a commercial radioimmunoassay kit (Cisbio International; Gif-sur-Yvette, France). The principle of the assay is based on the competition between the labelled cortisol and cortisol contained in calibrators or samples to be assayed for a fixed and limited number of antibody binding sites bound to the solid phase (coated tubes). Briefly 150 µl of calibrators, controls or samples were dispensed into the labelled coated tubes and 500 µl of ^125^I-cortisol was added to each tube. After incubation for 30 minutes at 37°C, unbound tracer was removed by a washing step with 1 ml of distilled water. The remaining radioactivity bound to the tubes was measured with a gamma scintillation counter calibrated for 125 Iodine. The amount of labelled cortisol bound to the antibody was inversely related to the amount of unlabelled cortisol initially present in the sample. Concentration of cortisol in saliva was determined by referring to the radioactivity of the 8-point calibration curve. The range of reference values for the morning and evening salivary cortisol concentrations at the CHU of Grenoble are 6.2–38 nmol/l at 06∶00–08∶00 AM, 0.8–4.9 nmol/l at 06∶00–08∶00 PM and <3 nmol/l at 10∶00–00∶00 PM.

**Figure 2:**
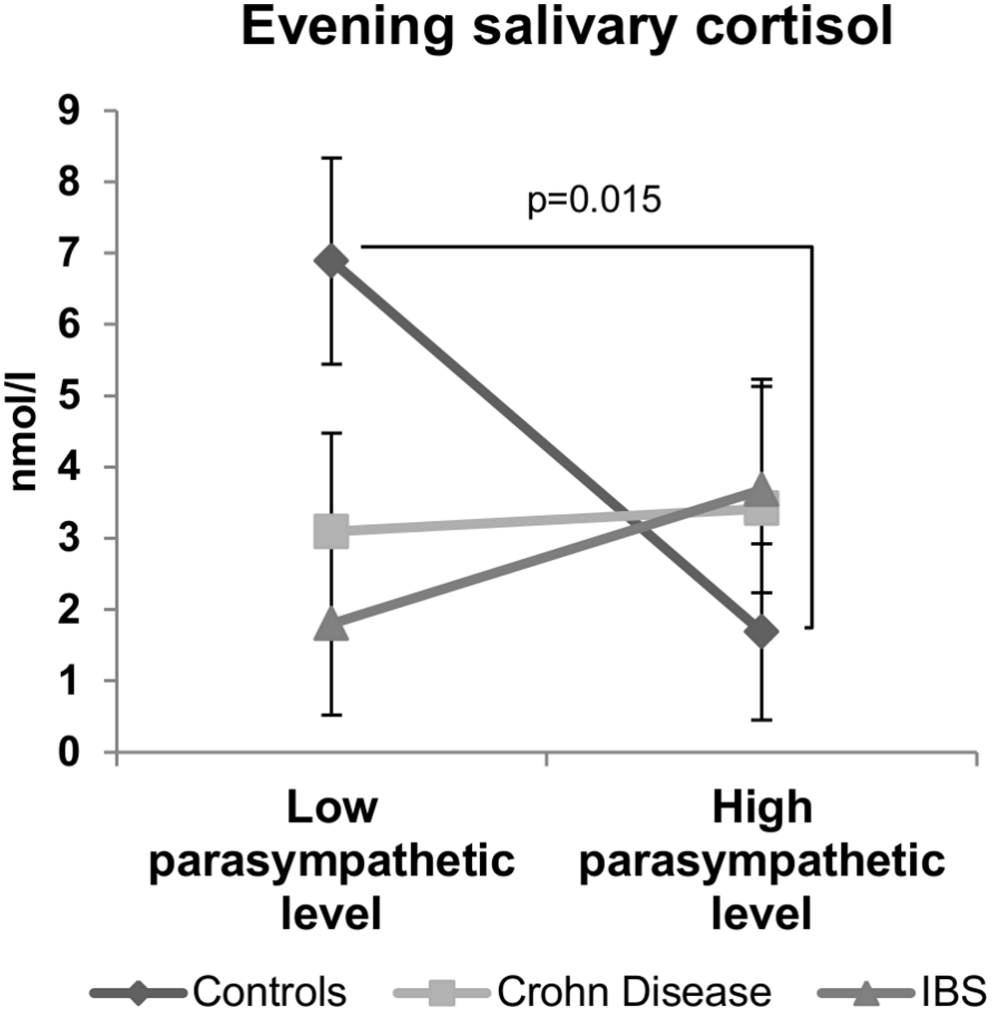
Relationship between the resting parasympathetic vagal tone and the evening salivary cortisol in controls, Crohn’s disease (CD) and Irritable bowel syndrome (IBS) patients. A balance was observed between the parasympathetic tone and the evening salivary cortisol in healthy subjects (control group) but not in CD and IBS patients. Data are expressed as mean ± sem. Comparisons are made between the high and low parasympathetic level subgroups using permutations test.

### Catecholamines Measurement

Analysis of catecholamines (epinephrine and norepinephrine) was performed with a commercial kit according to the manufacturer’s specifications (Chromsystems, Munich, Germany). Briefly, according to Hue [41], catecholamines were purified from plasma through solid phase extraction by aluminium oxide and secondly measured by reversed phase HPLC on isocratic mode with electrochemical detection (ESA-CoulArray, Eurosep Instruments, Saint Chamond, France).

### Psychological Assessments

*Anxiety* was assessed using the State-Trait Anxiety Inventory (STAI; [30], validated in French by Bruchon-Schweitzer and Paulhan [42] consisting of a scale with 20 items with a score varying from 20 to 80. A high score indicates high anxiety. In the present sample, the internal consistency was high (alpha = 0.91).

*Depressive symptomatology* was assessed by the Center for Epidemiologic Studies-Depression Scale (CES-D) [31], [32]. This brief scale of 20 items assesses symptoms or behaviours often associated with depression. The total score varies from 0 to 60, a high score signifying a high level of depressive symptomatology. An alpha coefficient for internal consistency of 0.85 has been reported in general population samples and 0.90 in psychiatric samples [43]. In the present sample, the alpha coefficient was 0.87.

**Figure 3:**
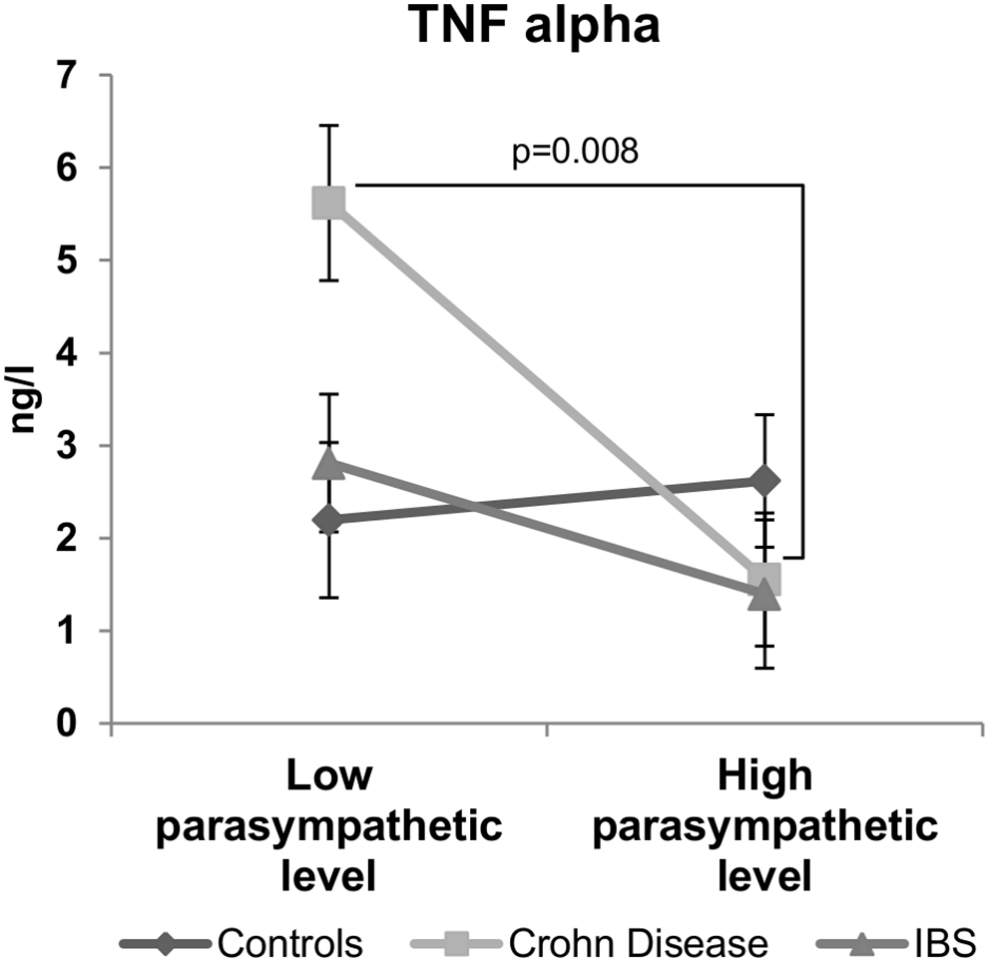
Specific inverse relationship between the resting parasympathetic vagal tone and TNF-alpha plasma level in CD patients. CD patients with low parasympathetic vagal tone exhibit a higher level of TNF-alpha than those with high parasympathetic vagal tone. This inverse relationship was not observed in controls or IBS patients. Data are expressed as mean ± sem. Comparisons are made between the high and low parasympathetic level subgroups using permutations test.

**Figure 4:**
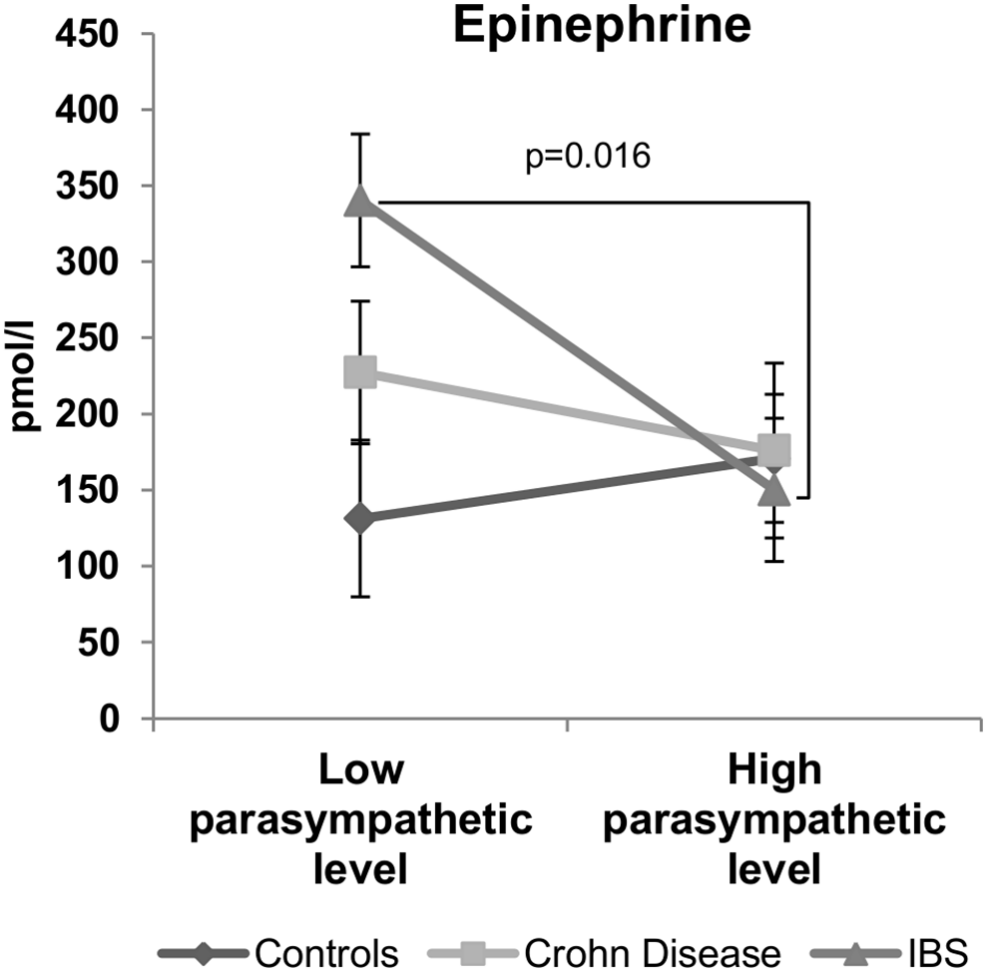
Specific inverse relationship between the resting parasympathetic vagal tone and epinephrine plasma level in IBS patients. IBS patients with low parasympathetic vagal tone exhibit a higher level of plasma epinephrine at rest than those with high parasympathetic vagal tone. This inverse relationship was not observed in controls or CD patients. Data are expressed as mean ± sem. Comparisons are made between the high and low parasympathetic level subgroups using permutations test.

### Statistical Analyses

Statistical analysis was carried out with Statistica 7.1 (Statsoft, Maisons-Alfort, France) and StatExact 9 (Cytel, Paris, France). ANOVA was used to evaluate main effects of group on age, disease duration, visceral pain, state anxiety and depressive symptomatology. When a significant effect was observed, a Bonferroni *post-hoc* test was applied to determine the differences between each group. Since the high frequency component of HRV is explained for 90% by the parasympathetic activity as described above, the normalized unit of HF (HFnu) has been considered to be the most appropriate, among HRV components, to represent the resting parasympathetic tone. Thus, HFnu was used to categorize subjects in high or low parasympathetic tone using K-means clustering method based on observations. Two clusters of subjects were therefore identified. Non-parametric permutation tests for small samples were performed to make comparisons between the low and high vagal tone subgroups within each group. Spearman correlation coefficients were used to evaluate relationships among vagal tone and cytokines or catecholamines within each group (controls, IBS and CD). Data are expressed as means (± standard error of the mean, SEM). The alpha value for statistical significance was set at p<0.05.

## Results

### Participants

Patients and healthy controls demographics and psycho-immunological data are detailed in table 1. Seventy-three subjects were distributed as healthy volunteers (controls), IBS and CD patients in remission. The mean age of all the participants was 38±10 years old. There was no significant difference in the age (F(2,70) = 0.85, p = 0.43) between groups. Among the 26 IBS patients, 7 patients (6 women and 1 man) were diarrhea predominant, 1 patient (woman) constipation predominant and the other 18 patients with alternative diarrhea/constipation. The mean duration of the disease was not significantly different between patients groups (F(1,45) = 1.46, p = 0.23). CRP plasmatic level was normal (<5 mg/l) in all groups. There was a significant effect of the disease on the level of perceived visceral pain as evaluated on the day of the experiment (F(2,70) = 7.48, p = 0.001). IBS patients had the highest score of perceived visceral pain compared to controls (p<0.001). There was also a significant effect of the disease on the scores of state-anxiety (F(2,66) = 7.63, p = 0.001) and depressive symptomatology (F(2.66) = 14.28, p<0.001) with CD and IBS patients exhibiting the highest scores of state-anxiety (p<0.05 and p = 0.001 respectively) and depressive symptomatology (p = 0.07 and p<0.001 respectively) compared to controls. Moreover, the scores of depressive symptomatology were significantly (p<0.02) higher in IBS than CD patients.

**Table 1:** Socio-demographic and psycho-immunologic data of the healthy control subjects, Crohn’s disease (CD) and irritable bowel syndrome (IBS) patients who participated to the study.

	Controls	Crohn’sDisease (CD)	Irritable BowelSyndrome (IBS)	p value
**Total number of subjects**	26	21	26	
**Mean age**, year ± SD	36±10	40±11	38±11	NS CD or IBS vs controls
**Sex**, M/F	8/18	9/12	7/19	
**BMI** (Kg/m^2^)	23±3.5	22±4.3	22±5.2	NS CD or IBS vs controls
**Mean duration of disease**,year (range)	-	13.4 (1–28)	10.3 (1–31)	
**Localization of Crohn’s** **disease according to** **Montreal classification**		***Ileal:***		
		L1B1: n = 3		
		L1B2: n = 3		
		B1pB3: n = 1		
		***Colonic:***		
		L2B1: n = 6		
		L2B1pB3: n = 2		
		***Ileocolonic:***		
		L3B1: n = 2		
		L3B2: n = 2		
		L3B2pB3: n = 2		
**Inflammatory markers (circulating levels)**				
**CRP level** (mg/l)	<4	<5	<5	NS CD or IBS vs controls
**Perceived abdominal visceral pain**				
**VAS**	0.30±0.34	1.28±0.38	2.19±0.34	IBS vs controls p<0.001
**Mood variables**				
**State-Anxiety**	31±1.90	39±2.15	41±1.91	CD vs controls p<0.05; IBS vs controls p<0.001
**Depressive symptomatology**	8.94±1.39	13.68±1.58	19.51±1.40	CD vs controls p = 0.07; IBS vs controls p<0.001; IBS vs CD p<0.05

### Balance between resting vagal tone and cortisol, TNF-alpha, epinephrine and negative affects in CD and IBS patients

#### The parasympathetic fingerprint

The HRV variable HFnu was used to categorize subjects into low and high parasympathetic tone as a hallmark of the level of their vagal tone. Two clusters of subjects were therefore identified as high or low parasympathetic level within control, CD, and IBS groups. This subgroup classification revealed that about half of the subjects had a high resting parasympathetic tone (HFnu = 56±1.5, n = 35) and the other one a low resting parasympathetic tone (HFnu = 25±1.5; n = 38). Data reporting mean values of HRV variables in low and high subgroups in controls, CD and IBS patients are detailed in table 2.

**Table 2:** Data representing the sub-group categorization based on HFnu-HRV K-mean classification.

	Controls		Crohn’s Disease (CD)		Irritable Bowel Syndrome (IBS)	
Resting parasympathetic level	High (n = 15)	Low (n = 11)	High (n = 8)	Low (n = 13)	High(n = 12)	Low (n = 14)
**HR (bpm)**	68±2	65±3	71±4	65±3	64±2	66±2
**RRI (ms)**	894±35	928±41	879±58	938±45	940±30	912±28
**Total Power (ms^2^)**	982±134	718±157	492±184	973±134*	885±150	693±140
**VLF (ms^2^)**	323±65	275±76	202±89	493±70**	387±73	311±67
**LFnu**	39±3	68±3***	36±6	63±4**	34±3	66±3***
**HFnu**	57±2	27±3***	56±3	20±3***	57±2	28±2***
**LF/HF**	0.74±0.2	2.75±0.2***	0.71±0.8	3.89±0.6***	0.62±0.3	2.79±0.3***

Data are expressed as mean ± sem. Comparisons are made between low and high parasympathetic level using permutations test.

*p<0.05;

**p<0.01;

***p<0.001.

Interestingly, CD patients with low parasympathetic tone showed significantly higher levels in Total Power (p<0.02) and VLF (p<0.01) HRV variables compared to CD patients with high parasympathetic tone. VLF seemed to be related to visceral sensitivity since (i) CD patients with low parasympathetic tone reported higher scores of perceived abdominal pain than CD patients with high parasympathetic tone (1.76±0.4 and 0.50±0.5 respectively; p<0.05) and (ii) VLF was positively correlated with the score of perceived abdominal pain (r = 0.65; p<0.001). It is interesting to note that this correlation observed in CD was not found in controls (r = –0.29; p = 0.14) or IBS patients (r = 0.30; p = 0.13).

#### Vagal tone and evening salivary cortisol level (figure 2)

Controls with high parasympathetic level (HFnu = 57±2) exhibited significantly (p<0.05) lower evening salivary cortisol (1.69±1.30 nmol/l) than controls with low parasympathetic level (HFnu = 27±3; evening salivary cortisol = 6.89±1.30 nmol/l). Interestingly, this inverse balance between morning vagal tone and evening salivary cortisol level was observed neither in CD (3.41±1.81 nmol/l for high parasympathetic tone and 3.09±1.38 nmol/l for low parasympathetic tone subgroup; p = 0.16) nor in IBS patients (3.68±1.44 nmol/l for high parasympathetic tone and 1.80±1.28 nmol/l for low parasympathetic tone subgroups; p = 0.42). In another way, it is interesting to note that no significant difference was observed between the high and low parasympathetic vagal tone subgroups for the morning plasma and salivary cortisol levels in any group (table 3).

**Table 3:** Influence of the vagal tone on the plasma levels of the morning salivary and plasma cortisol, IL-6, norepinephrine concentrations, state-anxiety and depressive symptomatology scores in Controls, Crohn’s disease (CD) and Irritable Bowel syndrome (IBS) patients.

	Controls		Crohn’s Disease (CD)		Irritable Bowel Syndrome (IBS)	
Resting parasympathetic level	High (n = 15)	Low (n = 11)	High (n = 8)	Low (n = 13)	High (n = 12)	Low (n = 14)
**Morning salivary cortisol (nmol/l)**	14.35±2.27	9.75±2.56	9.37±3.21	15.80±2.45	14.30±2.56	16.69±2.36
**Morning plasma cortisol (nmol/l)**	389.5±61.4	343±69.2	484.9±81.2	419.33±66.3	344.5±66.3	319.1±61.4
**IL-6 (ng/l)**	0.83±0.28	0.22±0.32	0.50±0.38	0.75±0.31	0.61±0.31	0.65±0.29
**Norepinephrine (pmol/l)**	1.8±0.18	1.6±0.22	2.3±0.24	2.05±0.2	2.01±0.20	2.38±0.19
**State-anxiety score**	33.06±2	29.1±3	37.7±4	40.2±2	41.1±3	41.3±2
**Depressive symptomatology score**	8.5±2	9.3±2	13.7±2	13.6±2	20.3±2	18.7±2

Data are expressed as mean ± sem. Comparisons are made between low and high parasympathetic level using permutations test.

#### Vagal tone and pro-inflammatory cytokines (figure 3)

In CD patients, a significant inverse relationship (r = –0.48; p<0.05) was observed between the parasympathetic tone and TNF-alpha plasma concentration. Thus, CD patients exhibiting a high parasympathetic tone (HFnu = 56±3) had significantly (p<0.01) lower levels of TNF-alpha plasma concentration (1.55±0.98 ng/l) than those with low parasympathetic tone (HFnu = 20±3; TNF-alpha = 5.62±0.80 ng/l). Such a negative correlation was neither observed in IBS patients (r = –0.34; p = 0.09) nor in controls (r = 0.19; p = 0.33) where the TNF-alpha plasma levels did not differ according to the parasympathetic vagal tone. As presented in table 3, IL-6 plasma levels measured in controls, CD and IBS patients were not different between the low and high parasympathetic vagal tone subgroups.

#### Vagal tone and catecholamines (figure 4)

In IBS patients, a significant inverse relationship (r = –0.39; p<0.05) was observed between the parasympathetic tone and the epinephrine plasma concentration. IBS patients exhibiting a high parasympathetic tone (HFnu = 57±2) had significantly (p<0.05) lower levels of epinephrine plasma concentrations (150±47 pmol/l) than those with a low parasympathetic tone (HFnu = 28±2; epinephrine = 340±43 pmol/l). Such a negative correlation was neither observed in CD patients (r = –0.07; p = 0.75) nor in controls (r = –0.05; p = 0.82). Norepinephrine plasma levels did not present any significant difference between high and low parasympathetic tone subgroups in control, CD and IBS patients (table 3).

#### Vagal tone and negative affects

No significant difference was observed between low and high parasympathetic tone subgroups for state-anxiety and depressive symptomatology scores within any group (table 3). However, in CD group, there was a significant correlation between evening salivary cortisol level and state-anxiety score (r = 0.49; p<0.05) on the one hand, and depressive symptomatology (r = 0.69; p<0.001) on the other hand. Such an association was not observed in IBS group.

## Discussion

The present study shows three important results highlighting the strong relationship between the vagal tone and markers of stress regulation and inflammation in CD and IBS patients. *First*, we observed that a high morning vagal tone is associated with a low evening cortisol level in healthy subjects but not in CD and IBS patients suggesting an uncoupling between vagal tone and cortisol level in those patients. *Second*, we found that TNF-alpha plasma level is negatively correlated to vagal tone in CD patients suggesting that the cholinergic anti-inflammatory pathway may be blunted in CD patients with low vagal tone. *Third*, we show that IBS patients with low vagal tone exhibit high plasma level of epinephrine as a mark of an unadapted high sympathetic activity. Finally, even if one limitation of our study may concern the possible impact of gender on the main effects that we observed, data reported here highlight the interest of measuring the resting vagal tone in IBS and CD patients as a marker of homeostatic imbalance that could predict a state of vulnerability to relapse.

### The resting vagal tone as a marker of the central homeostatic balance

In the present study, we have categorized individuals according to their resting vagal tone based on the HRV high frequency component (HFnu). The resting vagal tone is strongly involved in the regulation of physiological systems that are important in health and disease and notably those concerning the HPA axis and inflammation [23]. HRV has been previously proposed as an endophenotype marker particularly as a mediator between physiology and behavior [44]. In the present study, we used vagal tone rather like a fingerprint reflecting the balance of the autonomic network. Indeed, Thayer and Lane [44] described a model of neurovisceral integration in which a set of neural structures involved in cognitive, affective, and autonomic regulation referred as the central autonomic network or CAN [45] are related to HRV; thus they proposed HRV as an indicator of CAN-ANS integration. In this integrative interplay, the functional coupling between low cortisol levels and high vagal tone at rest would reflect, at the peripheral level, the central top-down inhibition of the medial prefrontal cortex on subcortical sympatho-excitatory circuits such as the amygdala [23], [46]. The hypoactivity of the medial prefrontal cortex enhances amygdala activity and then induce a parasympathetic withdrawal and a sympathetic activation. Thus, according to this model, the lower the vagal tone, the less active the prefrontal cortex will be, reflecting a shift from a homeostatic state to a stress state. This must be associated with emotional and physiological outputs such as an increase in pro-inflammatory cytokines, epinephrine and anxiety. In the present study, we have observed a negative coupling between the vagal tone and cortisol level in healthy subjects. Individuals exhibiting high resting vagal tone in the morning will have the greater decrease in salivary cortisol levels in the evening. In contrast, this balance between cortisol and HRV (vagal tone) was no more observed in CD and IBS patients. This argues for an uncoupling between the HPA axis and the ANS in both diseases and suggests a breakdown of the functional connectivity between the prefrontal cortex and the amygdala as recently shown in depression and anxiety [47], [48]. These results are independent of the circadian cycle since the salivary level of cortisol is high in the morning and low in the evening in the three groups as normally observed [49], [50]. According to the McEwen model of stress [11], this uncoupling would be the sign of a costly allostatic regulation with reduced flexibility in the regulatory systems. Such a situation would make CD and IBS patients more reactive to stressful life events or other challenging situations and thus more probable to trigger symptoms. Indeed, a reduced vagal tone could not be in favor of a positive effect of the cholinergic anti-inflammatory pathway (CAP) and thus inflammation and/or pain could be enhanced.

### Inverse relationship between vagal tone and TNF-alpha and perceived visceral pain in CD

The second important result reported in this study concerns the inverse relationship between HRV variables representative of the vagal tone and TNF-alpha in CD patients. TNF-alpha is a key pro-inflammatory cytokine in the pathogenesis of CD [51]. TNF-alpha is abundantly expressed in the gastrointestinal tracts of CD patients and contributes to intestinal mucosal inflammation [52]. Currently, the gold standard therapy aims at reducing the activity of TNF-alpha in IBD patients using anti-TNF therapies [53]. The vagus nerve is known to play a dual inhibitory control on inflammation. Its afferent fibers reach the brainstem and activate the HPA axis and cortisol release as an endpoint [4]. Further, more recently, vagal efferent fibers have been shown to exert an anti-inflammatory effect (i.e., the CAP) by inhibiting TNF-alpha production from macrophages [54]. Today, the CAP is a therapeutic target in chronic inflammatory diseases such as CD in which low frequency vagus nerve stimulation is used [55], [56]. Recent data, supporting our findings, have described an inverse relationship between HRV indices and TNF-alpha levels in heart failure [57] but also in healthy subjects under stressful situations [18]. In a recent review, Huston and Tracey supported the idea that HRV would be a relevant marker of excessive inflammation [58] in line with our findings. However, in our study, we did not found a significant relationship between HRV and TNF-alpha in healthy volunteers; a similar observation has also been recently reported in patients with chronic heart failure [59]. This is explained by the facts that healthy subjects (i) were under resting and not stressful conditions and (ii) were not under an inflammatory state.

The other interesting result of our study concerns the correlation between the spontaneous visceral abdominal pain perception and the VLF band of HRV. CD patients with low parasympathetic tone had higher levels of VLF than CD patients with high parasympathetic tone, and also reported higher scores of visceral perception. Such an observation has never been reported before. Although the physiological meaning of VLF oscillations has not been completely understood yet, the increase of this HRV variable is related to an important parasympathetic impairment with a loss of coherence between RR intervals and systolic blood pressure variability [60]. Furthermore, VLF power level is influenced by the renin-angiotensin system since the blockade of the angiotensin converting enzyme has been shown to decrease VLF [61]. In another way, VLF oscillations have been related to an increase in peripheral chemosensitivity in patients with congestive heart failure [62]. Angiotensin also acts as a modulator in the spinal transmission of nociceptive information [63]. Interestingly, a recent pilot study revealed an up-regulation of the renin-angiotensin system in inflammatory bowel disease patients [64]. Consequently, one can hypothesize that the increase of VLF oscillations observed in the low vagal tone CD patients, could be related to an impairment of the angiotensin system leading to the increase in visceral pain perception. This could enhance a shift toward hypersensitivity and IBS-like symptoms. If so, VLF oscillations would be a relevant marker of autonomic visceral sensitivity impairment that could be used in the patients’ follow-up. Further experiments are currently underway to deepen this question.

### Inverse relationship between vagal tone and epinephrine in IBS

Another important finding of our study is the inverse specific relationship between HRV and plasma levels of epinephrine in IBS. Patients with IBS exhibit visceral hypersensitivity (VHS) [65]. In our study, IBS patients reported higher scores of perceived abdominal pain than CD patients or healthy subjects. Besides pain, IBS patients reported more depressive symptoms and anxiety than healthy subjects. Psychosocial factors are often found in IBS patients and IBS is considered as a biopsychosocial model disorder [5]. Indeed, about 20 to 50% of IBS patients have psychiatric disorders, such as major depression, anxiety and somatoform disorders [66]. High perceived stress, negative mood and autonomic imbalance also characterized IBS as reported in previous studies [10], [67]. In the present work, we found that IBS patients exhibit higher circulating levels of norepinephrine at rest than healthy subjects. These findings are corroborated by several studies revealing abnormal catecholamines levels in IBS [68], [69]. In addition, our study reveals, for the first time, that IBS patients with low vagal tone have higher plasma levels of epinephrine than those with high vagal tone. This inverse relationship in addition to the uncoupling between the vagal tone and cortisol argues for a hyperactivity of the amygdala and a hypo-activation of the prefrontal cortex underlying vulnerability to stress in this disease [46]. This is strengthened by the elevated scores of state-anxiety and depressive symptomatology observed in those patients even if we did not find a linear relationship between the parasympathetic vagal tone at rest and these psychological scores. These affects would be rather associated to the HPA axis and thus to the level of cortisol as previously shown [70] and more probably to the decrease in the evening cortisol as suggested by the results of our study in CD patients.

## Conclusion

The fact that HRV is inversely related to TNF-alpha in CD patients and to norepinephrine in IBS, suggests that HRV would be a reliable marker of the allostatic load in such chronic diseases. This idea supports the fact that HRV that indexes vagal tone is a real marker of homeostasis and autonomic flexibility. In CD patients, the homeostasis of inflammation is imbalanced and a low vagal tone favors an overexpression of TNF-alpha. In IBS, a low vagal tone will be representative of a homeostatic imbalance of the sympatho-adrenergic axis. This is in agreement with the findings that in atherosclerosis, an inflammatory disease characterized by elevated levels of CRP and IL-6, a low vagal tone is inversely correlated with these inflammatory markers [71]. As we could see herein, among patients, only a part of them would require a vagal reinforcement that could be achieved by targeting the vagus nerve through electrical stimulation, pharmacology and/or complementary medicines such as hypnotherapy [9] or Mindfulness Based Stress Reduction a program which increases vagal tone [72]. These therapies would also improve visceral pain perception; reduce epinephrine and TNF-alpha levels allowing remission maintenance.
